# Isolation and characterization of nanocellulose from selected hardwoods, viz., *Eucalyptus tereticornis* Sm. and *Casuarina equisetifolia* L., by steam explosion method

**DOI:** 10.1038/s41598-022-26600-5

**Published:** 2023-01-21

**Authors:** Vishnu Raju, Revathi Revathiswaran, Kizhaeral Sevanthapandian Subramanian, Kalappan Thangamuthu Parthiban, Kalichamy Chandrakumar, Elaveetil Vasu Anoop, Cintil Jose Chirayil

**Affiliations:** 1 Forest College and Research Institute, Tamil Nadu Agricultural University, Mettupalayam, Tamil Nadu 641301 India; 2 Directorate of Research, Tamil Nadu Agricultural University, Coimbatore, Tamil Nadu 641003 India; 3 Department of Bioenergy, Tamil Nadu Agricultural University, Coimbatore, Tamil Nadu 641003 India; 4 Department of Chemistry, Newman College, Thodupuzha, Kerala India; 5 Present Address: Department of Forest Products and Utilization, College of Forestry, Kerala Agricultural University, Thrissur, Kerala 680656 India

**Keywords:** Chemistry, Materials science, Nanoscience and technology

## Abstract

Extraction of nanocellulose is challenging, especially from hardwoods due to its complex chemical structure as well as structural hierarchy. In this study, nanocellulose was isolated from wood pulp of two hardwood species, namely *Eucalyptus tereticornis* Sm. and *Casuarina equisetifolia* L. by steam explosion process. Pure cellulose wood pulp was obtained through Kraft pulping process followed by alkaline and bleaching pre-treatments. Isolated nanocellulose was characterized by Scanning Electron Microscopy (SEM), Transmission Electron Microscopy (TEM), Atomic Force Microscopy (AFM), Fourier Transformed Infrared (FTIR) Spectra, Thermogravimetric Analysis (TGA), and X-ray diffraction (XRD) studies. Nanocellulose obtained from both species showed non-significant difference with average diameter of 27.801 nm for eucalyptus and 28.690 nm for casuarina, which was confirmed from TEM and AFM images. FTIR spectra of nanocellulose showed prominent peaks corresponding to cellulose and absence of peaks corresponding to lignin. The elemental purity of nanocellulose was confirmed with EDAX detector. XRD analysis showed the enrichment of crystalline cellulose in nanocellulose, and also confirmed the significant conversion of cellulose I to cellulose II. During TG analysis the untreated fibres started to degrade earlier than the nanocellulose which indicated the higher thermal stability of nanocellulose. Highly entangled network like structure along with high aspect ratio make the nanofibres a versatile material for reinforcing the composites. This successful method can be replicated for industrial level production of cellulose nanofibres.

## Introduction

Advancement in science domains and subsequent emergence of new technologies led to the exploration of nanoscale materials of unique properties. Nanotechnology, a rapidly evolving area of science, engineering and technology made nanoscale materials a reality. In the beginning of the twenty-first century, the main focus of researchers, especially in the material sciences field, shifted to nanomaterials because of its unique multi-functional properties such as mechanical, thermal, magnetic, optical, chemical and biological properties. The properties of nanomaterials are usually different from bulk materials. Within a short span of time, the production of a good number of nanomaterials, such as, ZnO, TiO_2_, graphene, nanocellulose, nanochitin, nanotubes, were taken over from laboratory to industrial level production. Among these nanomaterials, nanocellulose is considered to have many desirable industrial and commercial properties and is produced hundreds of ton per year. Though the production of nano-fibrillated cellulose was patented in the early 1980s, its commercial production started very recently^1^. Global effort towards sustainable development led to the tremendous application of green, renewable and biodegradable materials to substitute non-renewable resources. In this way, nanocellulose also generated substantial interest globally. Cellulose nanofibres holds the major share of the nanocellulose market. Cellulose nanofibres held over half of the overall market shares in 2014^2^. Being an environment friendly, renewable, biodegradable, and sustainable organic material, nanocellulose is one of the best substitute for many non-renewable materials like petroleum products and viewed as important advanced biomaterials solutions in the pharmaceutical, cosmetic, packaging and composites markets^3^.

Nanocellulose is considered as the small fragments of cellulose chains having diameter in nanoscale. Literature on different forms of nanocellulose, its extraction, types of raw material used, properties and application, are widely available. Recent reviews^3–10^ explains various forms of nanocellulose that have been extracted using diverse cellulosic sources and distinct production processes. Various sources such as wood, herbaceous plants, grass, agricultural crops and their by-products, animal, algae and bacterial sources, waste paper, among others, are used as raw material to produce cellulose^11–21^. Wood is the most abundant source of cellulose, however, very few studies have been carried out to derive nanofibres from wood, especially from tropical hardwoods^11^. In this context, the present study was aimed at isolating and characterizing nanofibres from two hardwoods namely *Eucalyptus tereticornis* and *Casuarina equisetifolia*. These two species are prominent short rotation (3–8 years), fast growing pulpwood species in India widely used as raw material for paper manufacturing. Currently, mechanical methods (like homogenization^22,23^, cryo-crushing^24^, ultrafine grinding^25^, intense ultrasonication^26^, micro fluidization^27^, and high-speed blending^28^ and chemical methods (like acidic or alkaline treatment^29^, ionic liquid treatment, and 2,2,6,6-tetramethyl-piperidine-1-oxyl (TEMPO) mediated oxidation^30,31^ are widely employed for the extraction of nanocellulose. These methods require either higher energy (for mechanical methods) or hazardous chemical reagents (such as mineral acids which are corrosive in nature and hazardous to human beings and surrounding environment) which makes the most of the production process cumbersome. In contrast, steam explosion process is considered to be reliable, cost effective, efficient and environment friendly because of the use of low energy as well as the use of less harmful chemicals in low quantity. Steam explosion process had been previously used by Kaushik and Singh^32^ to isolate nanofibres from wheat straw; by Cherian et al.^33^ from pineapple leaf fibres; by Cherian et al.^33^ from banana rachis; by Abraham et al.^34^ from coir fibre; and by Deepa et al.^35^ from banana fibres. The present study is the first ever attempt to isolate nanofibres from hardwood pulp by steam explosion process. We hope that this model for the isolation of nanofibres can be replicated at the industrial level for the commercial production of nanofibres. (the terms nanocellulose and nanofibres are used interchangeably in the upcoming sections).

## Results and discussion

### Nanocellulose isolation process

Production of pure cellulose pulp is the first stage towards the production of cellulose nanofibres. Plant lignocellulosic fibres were modified during kraft pulping by the removal of non-cellulosic materials, swelling of crystalline region, and the elimination of hydrophilic hydroxyl groups. The basic lignocellulosic structure of wood was changed in reaction with alkali at very high temperature (165–170 °C). NaOH, a widely used chemical for pulping, was effective in eliminating the hemicellulose from the fibre. It is evident from Table 1. Due to reaction with NaOH, the hemicellulose fraction of wood underwent partial hydrolysis to produce sugars and the lignin component of wood depolymerized into phenolic compounds. The soluble sugars and phenolic compounds were removed from the medium by subsequent washing. The glycosidic linkages in hemicelluloses and ether linkages of lignin were hydrolyzed by acetic acid formed at high temperature from acetyl groups of hemicellulose (autohydrolysis)^36^.

**Table 1:** The chemical composition of untreated and treated wood fibres of two species namely *E. tereticornis* and *C. equisetifolia*.

Species	Treatment conditions	Cellulose (%)	Hemicellulose (%)	Acid insoluble lignin (%)
*E. tereticornis*	Untreated	46.09 ± 0.356	29.807 ± 0.994	22.10 ± 0.673
	Alkali treated	84.87 ± 0.683	7.177 ± 0.365	7.957 ± 0.677
	Bleached fibres	97.087 ± 0.160	< 1	1.233 ± 0.201
	Steam exploded	99	< 1	< 1
*C. equisetifolia*	Untreated	45.127 ± 0.110	30.853 ± 0.076	24.020 ± 0.183
	Alkali treated	84.08 ± 0.624	7.420 ± 0.656	8.501 ± 0.072
	Bleached fibres	96.03 ± 0.166	< 1	1.730 ± 0.098
	Steam exploded	99	< 1	< 1

In the second stage, partially delignified kraft pulp was treated with NaOH. During this process, hemicellulose and lignin fraction of raw fibre was dissolved out in alkaline medium^37^. Yamashiki et al.^38^ explained the chemistry of solubility of steam exploded cellulose in NaOH solutions. Intra-molecular hydrogen bond of the glucopyranose unit at the C3 and C6 positions is responsible for network structure and strength of cellulose chains. During the steam explosion in alkaline medium, partial breakdown of these intermolecular hydrogen bond at the C3 and C6 positions took place which changed the arrangement of macromolecular chains. Some loose substances present in fibre surface also has been removed during the alkaline steam explosion^36^. In the presence of NaOH, the carboxylic group of the pectin was ionized to form water soluble sodium carboxylate which was filtered off by continuous washing. This weakened the intermolecular hydrogen bond between the cellulose chains. Partial removal of lignin, hemicellulose, pectin, wax and other extraneous substances from fibre cell wall occurred during alkali treatment which exposed cellulose chains to further treatments. Alkali like NaOH under steam, depolymerizes the native cellulose chains, defibrillates the peripheral cellulose microfibrils and short length crystallites^35^. Lignin linked with carboxylate through two types of connections; alkali sensitive and alkali resistant. Ester type linkage between hydroxyls of lignin and carboxyls of uranic acid (which is derived from hemicellulose) is alkali sensitive linkage. Hydroxyl-hydroxyl connection between lignin and cellulose forms ether type linkage which is alkali resistant. Lignin degradation results in the formation of hydroxyls, carboxyls and carboxyls groups. These groups further facilitates solubilization of the lignin in alkaline condition and thus promotes cellulose purification^39^.

Third stage was the bleaching process. Bleaching resulted in degradation of residual lignin. Residual lignin is of a phenolic type which will not degrade during kraft cooking. The anion, i.e. ClO-derived from sodium hypochlorite will react with almost all the carbonyl groups of residual lignin to degrade them. During the bleaching treatment, the lignin reacts with NaClO_2_ to form lignin chloride which is then eliminated by continuous washing as it dissolves in the medium^40^. Bleaching also removes the remaining hemicellulose along with lignin. After bleaching, the cell walls of the individual fibres were separated, but the nanofibrillation never occurred.

**Figure 1:**
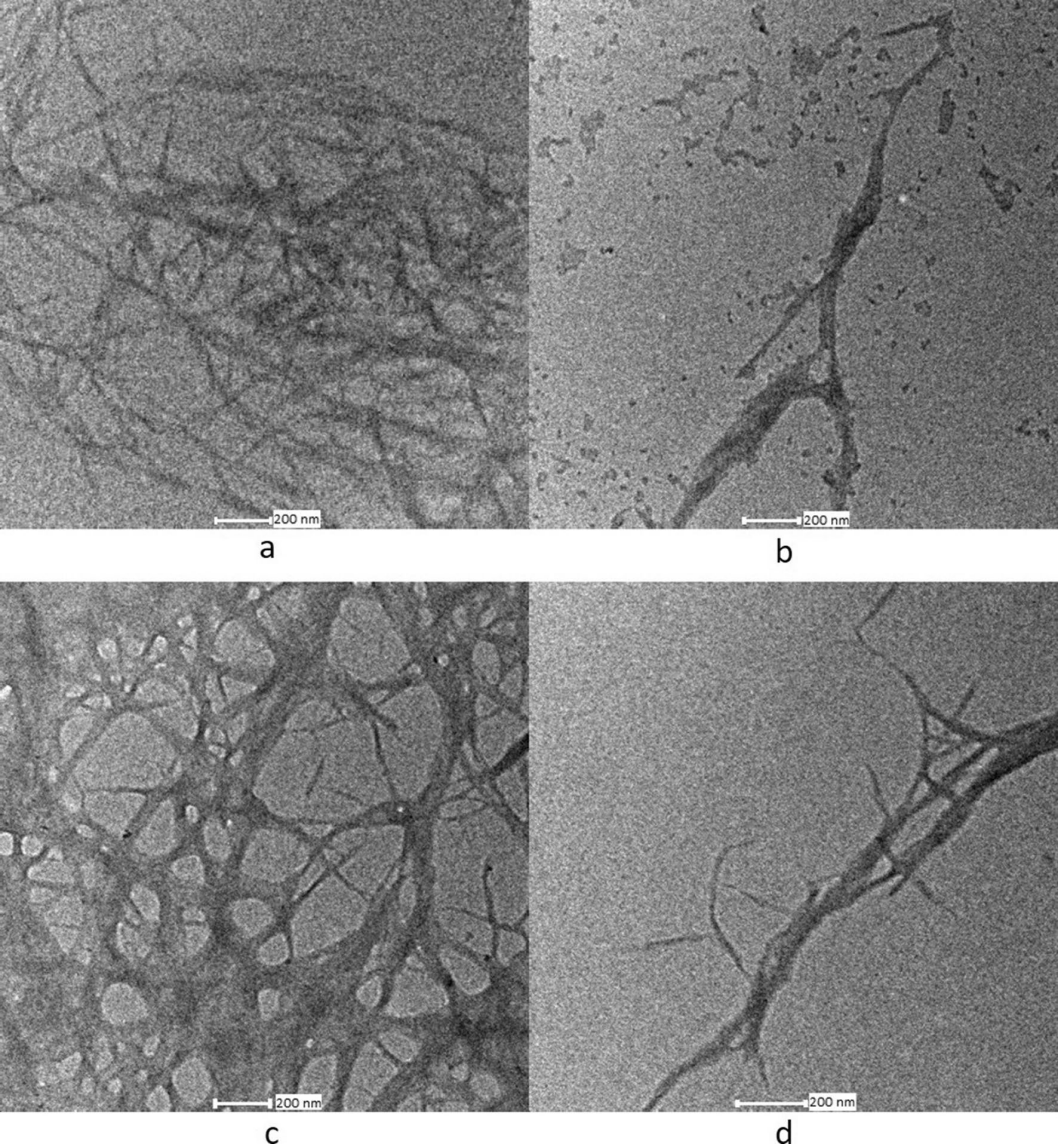
TEM images of (**a** and **b**) *Eucalyptus tereticornis* and (**c** and **d**) *Casuarina equisetifolia* wood nanocellulose.

Acid coupled steam explosion under high pressure separated the nanofibres from the fibre wall. Multiple steam explosion in acid medium under high pressure provided highly crosslinked nets like cellulose fibres (see the Fig. 1). During the period of the steam explosion process, the hemicellulose's glycosidic bonds were hydrolyzed, and the hemicellulose-lignin bonds were cleaved^39^. According to Xiao et al.^41^, in the presence of alkali like NaOH the α-ether linkages between lignin and hemicelluloses breaks lead to high solubility of lignin and hemicellulose. A rapid pressure drop in the vessel caused the fibres to expand or explode which defibrillates the cellulose chains or fibres. Bleaching of the alkali treated or mercerized fibres resulted in the complete removal of residual covering materials from the fibre. Acid hydrolysis followed by the steam explosion of bleached fibres produced cellulose nanofibres. The glycosidic bond connecting two anhydroglucose units hydrolyses when cellulose is acid hydrolyzed. As a consequence, acid hydrolysis dissolves the amorphous component, leaving the crystalline regions behind. Oxalic acid reaction with the weak regions of cellulose chains i.e. amorphous regions, resulted in the cleavage of cellulose chains to form nanofibrils. The amorphous regions of cellulose are more susceptible to chemicals such as alkalis, acids, etc. whereas the compact and stable network structure of crystalline regions are resistant to chemical reaction^42^. The pure cellulose slurry obtained after acid hydrolysis was further diluted into desired concentration and then dispersed well by using Ultra-Turrax T-50 homogenizer.

### Characterization of nanofibres isolated from *Eucalyptus tereticornis* Sm. and *Casuarina equisetifolia* L.

#### Visual analysis

**Figure 2:**
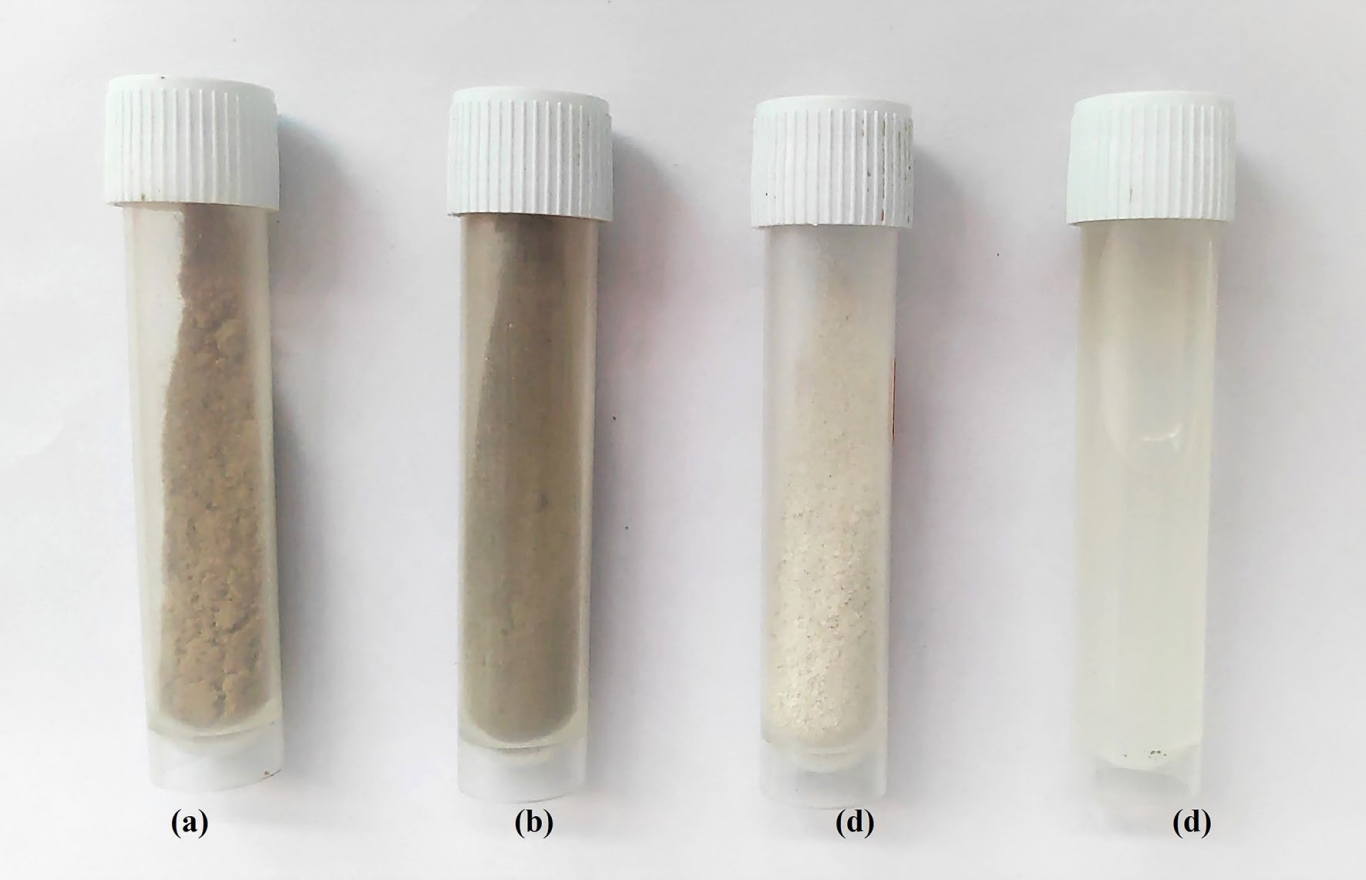
Powdered samples of (**a**) untreated wood (**b**) alkali treated fibres (**c**) bleached fibres and (**d**) nanofibres of *Eucalyptus tereticornis*.

Visual analysis of samples after each treatment were observed, including discoloration of pulp (see Fig. 2), and changes in structure and particle size. Initially, the wood was brown in color due to the presence of cellulose-hemicellulose-lignin matrix, then it became light brown and finally it turned to pure white. During the kraft pulp process, reaction of alkali at very high temperature eliminated the extractive substance and the other impurities like hemicellulose and lignin partially from wood. Kraft pulp produced was again treated with NaOH in the laboratory which resulted in the degradation of additional lignin and hemicellulose. Further, the bleaching treatment which was aimed to remove the lignin contents resulted in insoluble cellulose pulp residue having pure white colour. The structure of the samples changed from bulk wood chips to macro fibre bundles upon kraft process, followed by microfibers upon alkali and bleaching treatments and finally, the suspension of nanofibres network in water was formed. Visual appearance of the samples of both species after each treatment was similar throughout the process.

#### Morphological analysis using optical microscopy, SEM and TEM images

**Figure 3:**
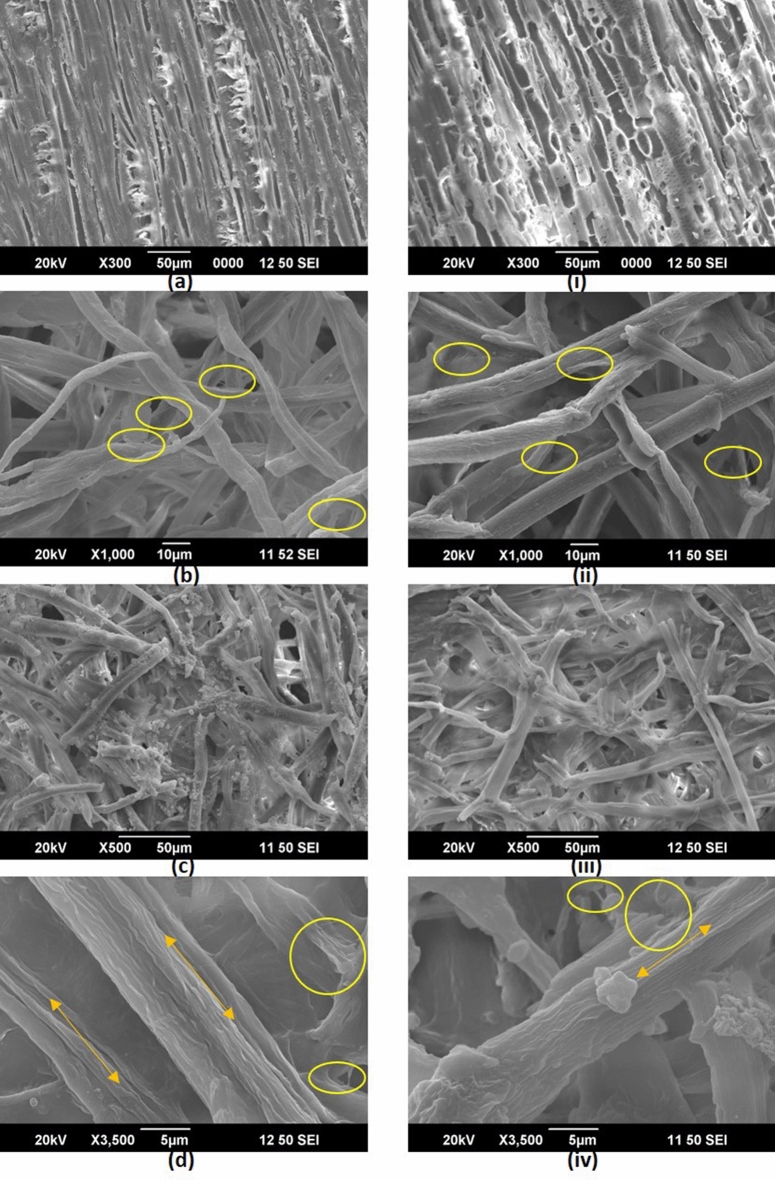
SEM images of (**a** and i) untreated fibres; (**b** and ii) alkali treated and bleached fibres; (**c**, **d**, iii and iv) oxalic acid treated steam exploded fibres (a-d are eucalyptus samples and i-iv are casuarina samples).

Optical and SEM micrographs of the wood sample of two species were taken to study the structure and size of the wood fibres (Fig. 3a). Untreated wood fibres were very stiff and compact with a spindle shaped structure. The cell wall of the untreated fibres were smooth because of the uniform distribution of lignin and hemicelluloses around cellulose microfibrils whereas the fibre wall surface of treated fibres were very rough with cracks and fissures and later defibrillated by subsequent alkaline and bleaching treatment.

Kraft pulping followed by alkaline treatment resulted in partial removal of the binding materials like hemicelluloses, lignin, pectin, wax and other impurities. Hemicellulose was hydrolyzed and became water soluble upon alkali-treatment which resulted in the initiation of the defibrillation process and the opening of the fibre bundles. In Fig. 3(b) and (ii), it can be seen that individual fibres were separated from bundles (in raw wood, fibre were in the form of bundles) to form a network like structure. Lignin is usually resistant to alkali treatment. Lignin acts as a natural cementing material which contributes the mechanical strength, stability, rigidity and durability to the cell walls. Even after alkali treatment the fibre maintained rigid compact structure and bundle form because of the presence of lignin which forms a bridge bond with the cellulose^43,44^.

Bleaching process resulted in complete removal of binding materials and all other impurities remained after alkali treatment. It resulted in reduction in diameter of the fibres. The initial diameter of wood fibres was 18.823 µm, and 22.725 µm, respectively for *Eucalyptus tereticornis*, and *Casuarina equisetifolia* which later decreased to less than 10 µm after successive chemical treatments (Fig. 3(c & d) and (iii & iv)). The aspect ratio (length by width ratio, (L/D)) of the fibres increased as a consequence of this reduction in size. For application as reinforcing material for polymers, the improvement in aspect ratio is certainly good. The complete removal of binding material by chemical treatment facilitated the fibre to defibrillate into microfibrils. Figure 3(c & d) clearly shows bleached microfibrils separated from fibres. Pure cellulose fibres obtained as a result of bleaching were long enough (hundreds of micrometers) to form network like structures. The next stage steam explosion process decreased its length which resulted in short fragments of the fibres (see Fig. 3(c, d, iii, & iv)). Later, the continuous and multiple times steam explosion resulted in the separation of the cellulose fibrils from the short fibres.

**Figure 4:**
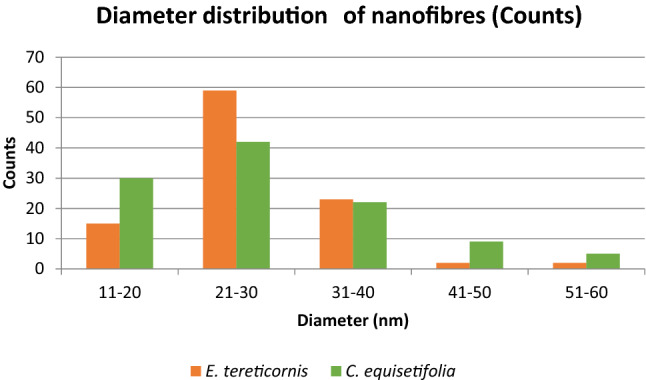
Diameter distribution of nanofibres extracted from eucalyptus and casuarina.

The steam explosion of bleached fibres in acid medium under high pressure converted microfibrils into nanofibrils (see Fig. 1(a, b, c and d)). High shear forces developed during sudden pressure drop resulted in the defibrillation of microfibrils. Length and diameter of the fibres were reduced by combined mechanical and chemical action. Length of the nanofibres obtained were not uniform because of the uncontrolled mechanical (shear) forces. Fibre diameter analysis showed both micro-and nano-sized fibres in the suspension. For both samples, diameter of the nanofibres ranged from 10 to 60 nm and the majority of the fibres were within the range of 20–30 nm (see Fig. 4) the average diameter obtained being, 27.801 nm and 28.690 nm for eucalyptus and casuarina respectively. The nanodimension of the fibres was confirmed with AFM images also (see Online Resource 1). Due to the entangled network like structure and difficulty in finding end points, the length of the fibres was not measured. However, nanofibres were found to have high aspect ratio. These nanofibers can be used as good reinforcing material in polymer matrixes in which the high aspect ratio of fibres will help to transfer the stress applied across the nanofiber-matrix interface. It will also improve the mechanical properties of the composite material^45^.

#### Chemical analysis

The results of chemical analysis of untreated and treated (Table 1) substantiated the results of FTIR. The chemical purity of cellulose nanofibres is vital because it will affect thermal, optical and other physical properties of the same. The cellulose percentage of eucalyptus and casuarina was increased after each treatment, i.e. after alkali and bleaching process. The dark brown colour of wood chips was due to the presence of high lignin content (Fig. 1). The colour of wood chips changed from dark brown to light brown due to partial removal of lignin after alkaline treatment. Partially delignified wood was bleached with sodium hydroxide and sodium hypochlorite for the complete removal of lignin and other impurities which was evident from white colour of the pulp. The data of chemical composition of fibre at each stage also substantiates this. Lignin content decreased from > 20% to < 1% for both the species.

#### FT-IR Spectroscopy analysis

**Figure 5:**
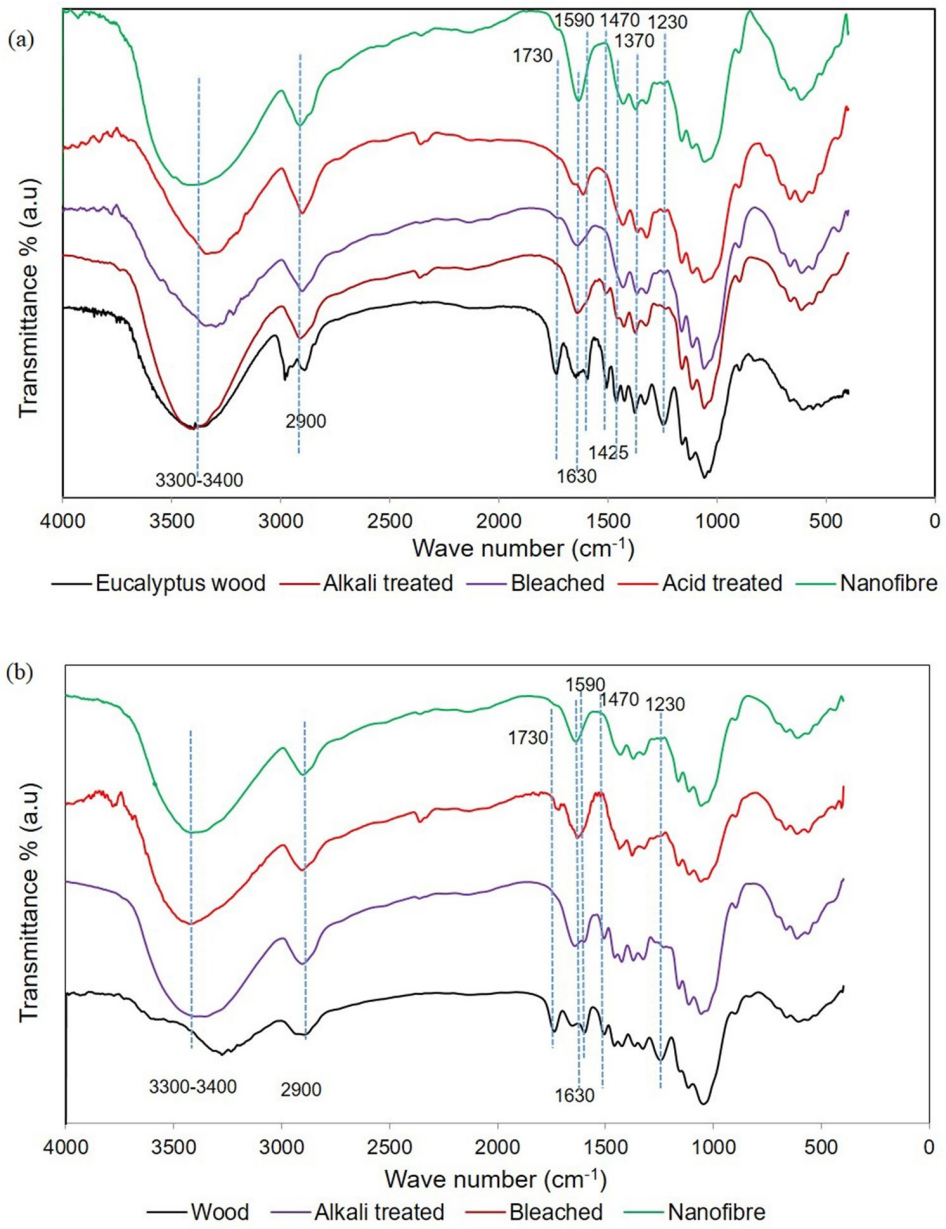
FT-IR spectra of untreated and treated samples of (**a**) *Eucalyptus tereticornis* and (**b**) *Casuarina equisetifolia*.

The FTIR spectra gives a quick overview of the ratio of lignin, cellulose, hemicellulose, pectin, aromatic and other compounds in wood^46^. FTIR spectroscopy can be effectively used for the comparison of samples for its chemical changes after each treatment. Chemical changes obtained in fibres at different stages, from untreated wood samples to nanofibres, was analysed from the spectra (Fig. 5). The infrared absorption spectra between 1800 and 600 cm^− 1^ was of particular interest for comparison between samples. This region provides distinctive molecular fingerprints corresponding to the chemical bonds of the lignocellulosic composites, including the O–H vibration, C–H deformation, and C–O stretch of cellulose, hemicellulose, and lignin, C–O–C vibration in cellulose and hemicellulose, and O–H vibration of the absorbed moisture.

The chemical changes obtained after each treatment was similar for both the species. In the spectra of fibrils, a number of peaks disappeared after successive treatments which indicated the elimination of hemicellulose, lignin, and other extraneous substances. The peaks at 875 cm^−1^, 1730 cm^−1^, and 1750 cm^−1^ represents glycosidic linkage, ketone/aldehyde C = O stretch and free ester linkages of hemicellulose^47^. Among these, peaks at 1730 cm^−1^ was very prominent in FTIR spectra of two wood samples. The FTIR peak at 1730 cm^−1^ of raw wood fibre was scribed to the C = O stretching vibration of the acetyl and uronic ester groups of either pectin or hemicellulose, or the ester linkage of the carboxylic group of lignin (ferulic and p-coumaric acids of lignin forms ester linkage) and/or hemicellulose^48,49^. Alkali and bleaching treatments completely removed the hemicellulose (along with pectin and waxes) from wood fibre which resulted in absence of peaks at 1730 cm^−1^ in the remaining FTIR spectra. According to Paul et al.^50^, the absence of band in nanofibrils at 1730 cm^−1^, (which is characteristic of hemicellulose polymer), confirms the complete elimination of hemicellulose during chemical pre-treatment.

Peaks attributed to lignin polymer are at 1215 cm^−1^, 1270 cm^−1^, 1327 cm^−1^, 1370 cm^−1^, 1425 cm^−1^, 1465 cm^−1^, 1500 cm^−1^, 1595 cm^−1^, 1682 cm^−1^, 2840 cm^−1^, 2937 cm^−1^ and 3421 cm^−1^. The assignment of absorption bands to particular molecular bonds or even specific chemical compounds is not always straightforward due to the ambiguity of biological samples and the overlap of absorption bands^51^. In the present study, prominent peaks of lignin were observed at 1230 cm^−1^ (≈1215 cm^−1^), 1425 cm^−1^, 1470 cm^−1^ (≈1465 cm^−1^), and 1590 cm^−1^ (≈1595 cm^−1^) in all the wood samples. Similar to hemicellulose, lignin peaks were absent in FTIR spectra of nanofibres because of complete removal of lignin after alkali and bleaching treatments. The aromatic skeletal vibration of lignin caused a peak at 1230 cm^−1^ in raw fibre, which was considerably reduced in treated fibres due to the removal of lignin by chemical treatments. The peaks at 1370 cm^−1^ in the spectrum attributed to the C–H deformation vibration in the aromatic ring of lignin which disappeared completely in the spectra of bleached fibres and CNFs^52^. Alkali treatment resulted in only a partial removal of lignin from wood which resulted in small peaks of lignin at 1470 cm^−1^ for all alkaline treated samples. The absorption peak between 1225–1250 cm^−1^ present in the raw fibre spectra is scribed to the C–O out of plane stretching vibration of aryl group in the lignin^53^. In the spectra of chemically modified fibres, this peak had vanished entirely.

The peaks related to cellulose were present in samples irrespective of treatments, but the peaks were very prominent in nanofibres. Many of the peaks in FTIR spectra were common to cellulose, hemicellulose and lignin polymers. For example, peaks at 1030 cm^−1^ represents C–O, C=C, and C–C–O stretching of cellulose, hemicellulose and lignin which was observed in all samples. For all samples, a wide band present in the region of 3400 to 3300 cm^−1^ indicates the free O–H stretching vibration of hydrogen bonded hydroxyl group. It represents the hydrophilic tendency of the samples. The peak at ≈2900 cm^−1^ was scribed to C–H stretching^24,49,54^. The most significant absorption band, which is present in all samples, even after alkali and bleaching treatment, is at 898 cm^−1^ which relates to glycosidic –C–H– deformation, with a ring vibration contribution and –O–H bending. These features are typical of the β-glycosidic linkage of anhydroglucose units in cellulose^55^. The bending vibration C–H and C–O bond in the polysaccharide aromatic rings of lignin complex is connected to the vibration peak found in all fibre samples between 1360 and 1375 cm^−1^^56^.

The absorbance peak seen in the spectra in the region between 1627 and 1638 cm^−1^ was ascribed to the O–H bending of the absorbed water^53^. A double peak was observed between 3000–2800 cm^−1^ for eucalyptus wood which was exceptional in comparison to the other wood sample. The peaks between 2920 and 2850 cm^−1^ might have originated from the asymmetric and symmetric stretching of methyl and methylene groups from organic extractives present in wood^57^. In this context, double peak observed at 3000–2800 cm^−1^ for eucalyptus wood might be due to the high amount of oil and extractives.

#### X-ray diffraction analysis

The x-ray diffraction graphs of both samples were quite similar, which showed a significant predominance of cellulose component. But the crystallinity index obtained from the two methods were different. Crystallinity index calculated by Segal method was comparatively higher than the values obtained from area method. Similar trend was observed by Park et al.^58^. They found that, in Segal method, the intensity of amorphous curve (I_am_ value) used for crystallinity index estimation is significantly underestimated, which results in overestimation of the CI. CI values calculated by area method was significantly low as compared to CI values obtained from intensity peak method. However, the trend was similar in both methods.

Chemical treatment of raw materials targeted to eliminate amorphous components, so that the crystalline region of the produced cellulose nanofibers becomes more evident^24^. Crystallinity index (CI) of samples was found to increase after successive stages (Table 2). Cellulose crystallinity is the key factor for determining their mechanical and thermal properties of the individual fibres. Increase in the CI would increase the mechanical properties and thermal stability of fibres. Inter- and intra-hydrogen bonding and Van der Waals forces between adjacent molecules provide the crystalline structure to cellulose, whereas the hemicellulose and lignin are amorphous in nature.

**Table 2:** Crystallinity index (%) of untreated and treated fibres of *E. tereticornis* and *C. equisetifolia*.

Species	Treatment conditions	Intensity method	Area method
*E. tereticornis*	Untreated	60.23	38.42
	Alkali treated	63.58	50.23
	Bleached fibres	80.87	56.85
	Nanofibres	82.31	60.97
*C. equisetifolia*	Untreated	60.38	42.54
	Alkali treated	74.87	54.97
	Bleached fibres	80.69	57.12
	Steam exploded	85.55	68.76

Chemical treatments like alkali treatment, bleaching and acid hydrolysis performed on the wood fibres can affect crystalline structure of cellulose. Therefore, sometimes the efficiency of the chemical treatment can be evaluated indirectly by measuring the crystallinity of chemically treated fibres. In this study, treated samples were more crystalline than the initial untreated wood fibres which was as evidenced from the decrease in or elimination of the peak due to the elimination of amorphous structures. Presence of amorphous constituents, mainly hemicellulose and lignin, would reduce the CI of fibres. Raw fibre possess the highest percentage of lignin and hemicellulose. By chemical treatment, i.e., alkali treatment and successive bleaching processes, these noncellulosic constituents were removed which resulted in increase in crystallinity of fibres.

**Figure 6:**
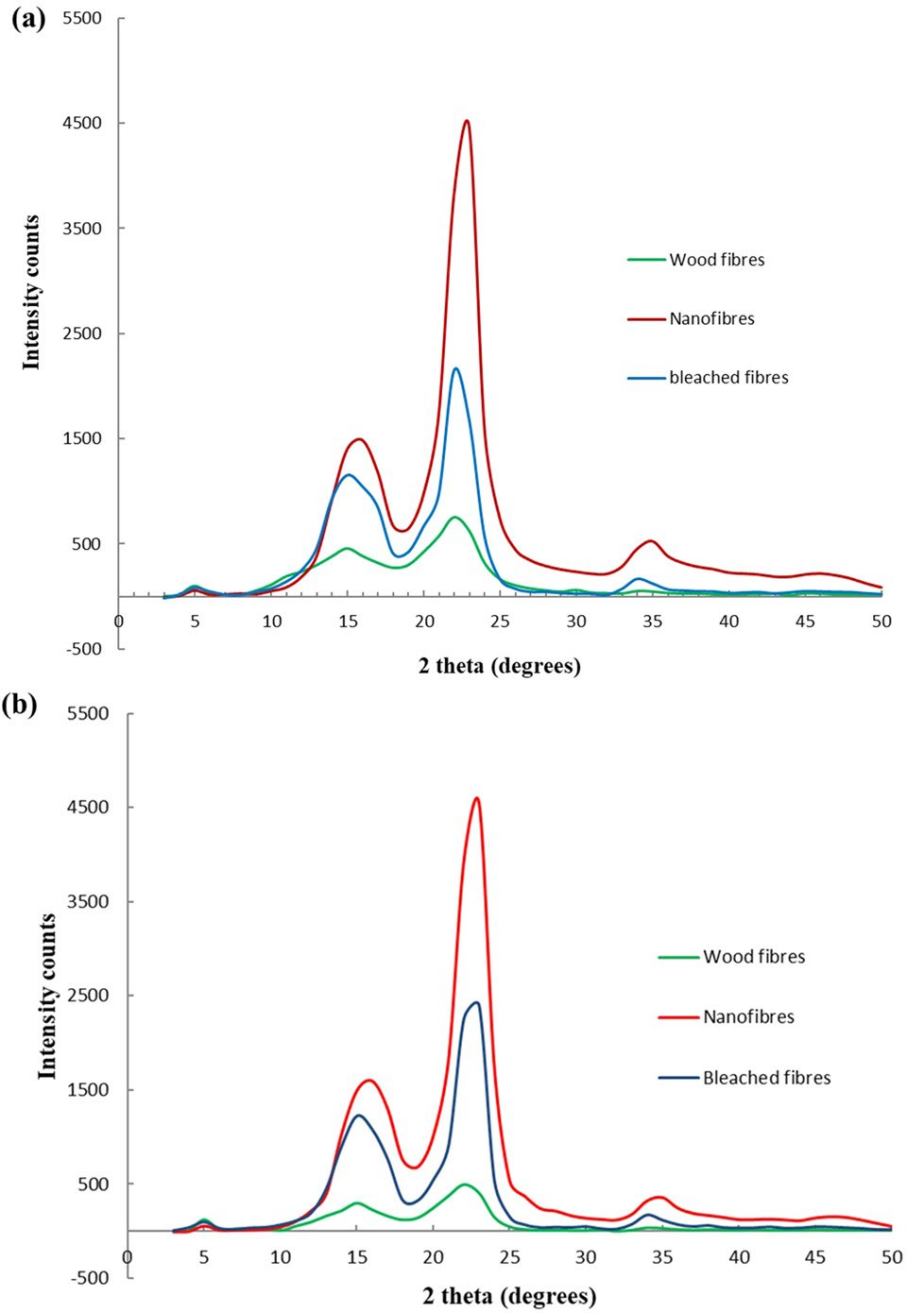
XRD spectra of untreated and treated samples of (**a**) *Eucalyptus tereticornis* and (**b**) *Casuarina equisetifolia*.

X-ray diffraction pattern drawn in Fig. 6 showed the changes in the crystallinity of fibres of two species after each treatment. Treated and untreated fibres exhibited three main reflection peaks at 2θ ≈15°, 22° and 34° corresponding to the (101), (002) and (040) crystal planes of cellulose crystalline structure, respectively. The minimum near 18° ascribed to amorphous wood regions^59^. The width and intensity of peaks at 2θ ≈15°, 22° and 34° represented the relative crystallinity of samples. The narrow and intense peaks was attributed to high crystalline nature of samples. The sharp narrow peak at 2θ ≈22° in the X-ray diffraction pattern of the acid treated fibres clearly indicated very high crystallinity due to more efficient elimination of non-cellulosic chemical components and dissolution of amorphous zones. The intensity of peak at2θ ≈15° was also relatively high for acid treated fibres as compared to fibres of previous stages. Significant increase in relative intensity of the peaks at 2θ ≈15° and 22° were observed giving rise to a pattern typical of cellulose I patterns. Similar results were obtained by Li et al. and Marchessault and Sundararajan^60,61^. Small peak at 2θ ≈ 34° was evident only for bleached and acid treated fibres of both species. Bleaching treatment removed almost all the lignin fraction of the fibres that resulted in the emergence of this peak.

#### TG analysis

**Figure 7:**
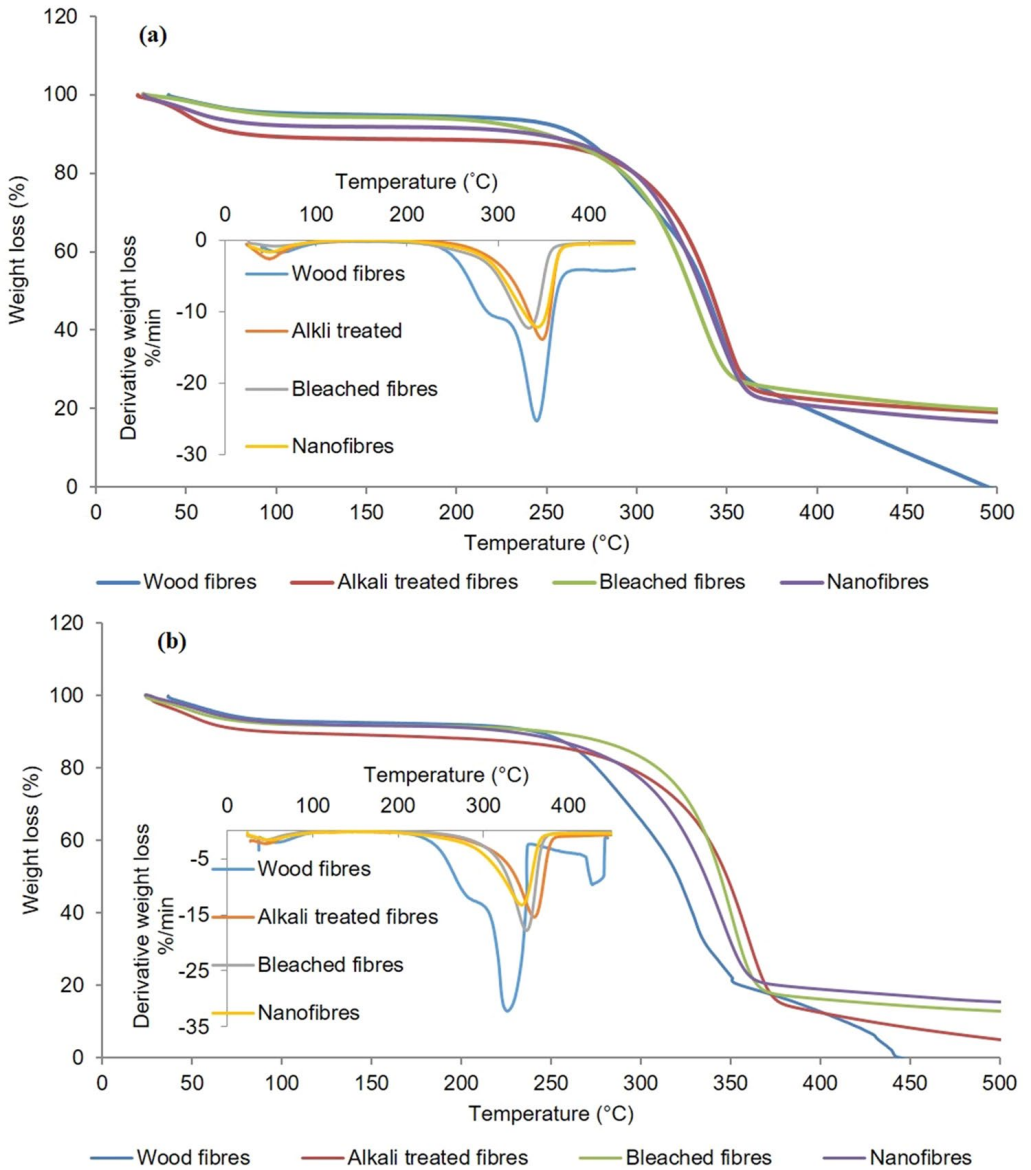
The per cent weight loss vs. temperature graph and DTG curve of untreated and treated samples of (**a**) *Eucalyptus tereticornis* and (**b**) *Casuarina equisetifolia*.

The degradation characteristics of fibre at different phases of preparation were analysed by thermogravimetric analysis (TGA). The per cent weight loss vs. temperature graph (Fig. 7) showed two weight loss regions for both treated and untreated fibres. The initial weight loss region between 80–150 °C was mainly due to the vaporization of free and bound water present in the sample^62^. A small trough on the left side of the DTG curve clearly indicates this. This weight loss was similar for both treated and untreated fibres. High amount of exposed hydroxyl groups on nanofibre surface resulted in more absorption of moisture or bound water. The next weight loss region was between 250–400 °C due to the thermal degradation of hemicellulose, lignin and the breakup of cellulose glycosidic linkages^63^. The broad peak in the region from 250–450 °C was contributed by the degradation of lignin components. The cellulose components start to degrade after hemicellulose and lignin. The major degradation process, which resulted from the depolymerisation, dehydration and decomposition of polymers occurred within different temperature range depending on the treatment^64^. The thermal stability of chemical components are in the order hemicellulose < lignin < cellulose. Thermal degradation of hemicellulose and cellulose occurred at 260 °C and 375 °C respectively. From the graph, it is clear that the major peaks of thermal degradation is different for treated and untreated fibres. Treated fibres showed more thermal stability than untreated fibre. Untreated fibre is composed of long cellulose chains or fibrils, which is surrounded by high concentration of lignin, hemicellulose, pectin and other impurities. Hemicellulose and lignin are arranged within and between cellulose fibrils and is intimately associated with the structure of cellulose. These chemical components may initiate more active sites and accelerate the beginning of thermal degradation so that raw fibre starts to degrade early than treated fibres. Nguyen et al.^65^ reported that the higher thermal stability of treated fibres is the result of complete removal of hemicelluloses and lignin from the fibres.

Onset degradation temperature (T_on_) and temperature at maximum degradation rate (T_d_) of untreated and treated fibres of both species is given in Table 3. T_on_ and T_d_ of treated and untreated fibres of casuarina showed similar trend in previous studies. However, in eucalyptus, exceptionally high T_d_ values for untreated samples and relatively low T_on_ and T_d_ values for bleached fibres was observed. Interestingly, alkali treated fibres of two species showed higher thermal stability (higher T_on_ and T_d_) than bleached fibres and nanofibres. This was a result of the compact structure of alkali treated fibres (not much defibrillated into microfibres) that reduced the active sites and area exposed to thermal degradation. Moreover, Nguyen et al.^66^ reported that the fractional removal of hemicellulose and lignin, the higher crystallinity of the cellulose and the compact bundle like structure of fibres would provide better thermal stability of fibres. Alkali treated fibres satisfied these criteria as well. The improvement of thermal stability of NFs was due to the absence of hemicelluloses, lignin and pectin which have a lower decomposition temperature compared to cellulose^67^. Decrease in thermal stability of bleached and nanofibres was related to reduction in particle size and defibrillation by chemical and mechanical treatments. The decrease in the thermal stability of cellulose fibres due to intensive mechanical and chemical treatments has already been observed by Chen et al.^68^ and Chowdhury and Hamid^69^. The higher surface area combined with a nanometric dimension and large number of free ends of cellulose chains were considered as the main cause for this behaviour. Huang et al.^70^ reported that the thermal stability of nanofibers prepared mechano-chemically from cassava residue was decreased due to the size reduction of the fibres to nanometric scale, increased specific surface area and increased numbers of exposed active groups. According to Yildirim and Shaler^71^, the reduced thermal stability of the highly crystalline cellulose chains attributed to its high thermal conductivity. The highly crystalline cellulose are efficient pathways for the heat transfer.

**Table 3:** Onset degradation temperature (T_on_) and temperature at maximum degradation rate (T_d_) of untreated and treated fibres of *E. tereticornis* and *C. equisetifolia*.

Species	Treatment conditions	Onset degradation temperature (T_on_) (°C)	Temperature at maximum degradation rate (T_d_) (°C)
*E. tereticornis*	Untreated	236.45	343.68
	Alkali treated	266.69	348.88
	Bleached fibres	236.03	334.64
	Nanofibres	255.89	343.85
*C. equisetifolia*	Untreated	224.86	327.90
	Alkali treated	261.16	360.28
	Bleached fibres	267.50	351.00
	Steam exploded	244.43	345.35

DTG curves of treated and untreated fibres clearly depicted the weight loss regions of fibres during thermal degradation. A trough at 40–100 °C is the first weight loss region which is due to loss of moisture of the sample. The second weight loss region is at 250–300 °C attributed to decomposition of hemicellulose and the slow degradation of lignin which is very prominent in raw fibres. Next weight loss region is at 215–300 °C, present in all fibres represent degradation of cellulose as discussed earlier. These results are consistent with previously published data on thermal degradation of raw fibres and nanocellulose^72–76^.

## Methods

### Materials

For the study, eucalyptus and casuarina trees were felled from the research plots established in Forest College and Research Institute, Mettupalayam (Lat. 11°19′25.37’’ and Long. 76°56′8.84’’), Tamil Nadu, India. The logs collected from the felled trees were chipped using a pilot chipper and then graded using vibrating chip classifier to get uniform sized (about 2–3 cm long) wood chips. Then the wood chips were subjected to washing to remove impurities. After washing the wood chips were dried under atmospheric conditions for 2 weeks and then stored in air tight container for further use. Chemicals such as Sodium hydroxide, Sodium carbonate, Sodium sulphide, Oxalic acid, Acetic acid and Sodium chlorite were obtained from Sigma-Aldrich, India. All the chemicals used were of Analytical Reagent grade (AR grade) and used as received.

### Methodology

#### Fibre morphology of wood

Jeffrey’s method was used to macerate wood fibres^77,78^. Jeffrey’s solution was prepared by mixing equal volumes of potassium dichromate and nitric acid solution having 10% concentration. Longitudinal wood shavings of minimum thickness were taken from dried wood chips using a razor blade. These shavings were taken in test tubes and then boiled in the Jeffrey’s solution for 5–10 min. After boiling, test tubes were kept for some time to cool and to settle down the fibres at the bottom. After avoiding the supernatant, the fibre residue at the bottom of the test tube was carefully washed with distilled water multiple times until the traces of maceration solution were removed. After washing, the fibres were transferred to watch glass and stained using safranin for 5 min. Then the stained fibres were mounted on glass slides using glycerin as the mountant. Microscopic examination and measurements of fibres on these slides were carried out using an Image Analysis system (Labomed-Digi 2). A total of 100 randomly chosen unbroken fibers were examined to measure fibre length and fibre diameter.

#### Chemical analysis of wood

For the chemical analysis, dried wood chips were first powdered using a Wiley mill. Wood powder sieved through a 40-mesh size but retained over 60 mesh was then subjected to chemical analysis for calculating percentage of α-cellulose, holocellulose and acid insoluble lignin. TAPPI standards such as T-203 for α-cellulose, T-249 for holocellulose, T-222 for lignin and T-204 for ethanol–benzene extractives, were followed for chemical analysis of treated and untreated samples^79^.

### Nanocellulose preparation process

#### Kraft pulping

The pulping was carried out in a 10 L stainless steel electrically heated rotary digester as per the TAPPI (Technical Association of Pulp and Paper Industry) method^79^. The chips (1 kg, oven dry basis) were filled into the digester and then the pulping liquor was added at 5:1 liquor-to-wood ratio (bath ratio). The chips were impregnated with white liquor for 20 min at 40 °C before the temperature was increased to a maximum of 165–170 °C. The time for cooking temperature and time at maximum temperature (165–170 °C) were kept as 90 min and 120 min respectively. The active alkali varied from 17.0% to 20.0% Na_2_O on chips, while sulfidity varied from 30 to 32%. After cooking, the digester was allowed to cool to room temperature. Cooked wood was defibrillated with beater for 20–30 min and then washed with water until all the traces of black liquor was removed. The washed pulp was stored in the form of solution and used whenever required.

#### Preparation of nanocellulose from wood pulp

The methodology for the preparation of nanocellulose was adopted from^64^. In brief, during the first stage of alkali treatment, pre-weighed partially delignified wood pulp was treated with 2wt% sodium hydroxide under 25 psi pressure and 115–125 °C temperature for 1 h in an autoclave. After releasing the pressure, the alkali process was continued and repeated for three times. Following this, the alkali treated wood pulp was treated with a bleaching solution. It was prepared by mixing equal volumes of 25% sodium hypochlorite solution and a mixture of acetic acid and NaOH (27 g and 78.8 g respectively in one litre) solution. The bleaching was repeated six times. Thoroughly washed bleached fibres were then subjected to steam explosion process in 10% oxalic acid medium under a pressure of 25 psi in an autoclave. The fibres were kept in acid medium under high pressure and temperature (120–130 °C) for 15 min. From the high-pressure level, the fibres were subjected to reach atmospheric pressure abruptly by sudden opening of valve and the process was repeated eight times. The fibers were washed thoroughly till it was acid free. Colloid like white mass obtained was then diluted with water and stirred well with a mechanical stirrer of type RQ-1.27 A at 8000 rpm for 4 h until the fibres were dispersed uniformly.

#### Characterization of samples

For the characterization, the study samples were classified into treated and untreated (or raw wood) fibres. Treated wood fibres include alkali treated, bleached and steam exploded fibres (or nanofibres).

#### Optical microscopy

It was done by an Image Analysis System which consisted of an optical microscope (Leica), a digital camera and a computer. The camera captured digital images from an optical microscope which were further analysed by the image analysis software ‘Labomed DigiProVersion 2.0’ (for software details, follow https://www.oem-optical.com/labomed-digiplus-digital-microscope.html).

#### Scanning electron microscopy

A scanning electron microscope (Jeol, JSM 6390LA) (Accelerating voltage: 0.5 to 30 kV, Filament: Tungsten, Magnification × 5 to 300,000) was used for the morphological characterization of samples at different stages of production process. EDAX detector (EDAX model OXFORD XMX N with resolution 136 eV, EDAX detector area 30 mm^2^) attached to an SEM was used for elemental analysis of the nanocellulose samples. The sample was smeared on a small piece of adhesive carbon tape which was fixed on a brass stub. The sample was then subjected to gold coating using sputtering unit (model: JFC1600) at 10 mA of current for 10 s. The gold coated sample placed in the chamber of the SEM and secondary electron/back scattered electron images are recorded.

#### Transmission electron microscopy

Transmission electron microscope (Jeol/JEM 2100) with (200 kV, LaB6 Electron gun, Point resolution 0.23 nm, Lattice resolution 0.14 nm) was used for the morphological characterization of the nanofibre. The samples were dispersed in water. The solution was then dispersed well using an ultrasonicator. A drop of the well dispersed solution was then pipetted out and the drop casted on copper micro-plates followed by evaporation of the solvent. After drying, it was fixed in the specimen holder.

#### Atomic force microscopy analysis

Surface morphology of cellulose nanofibres extracted from wood was analyzed using atomic force microscopy (Type: NanoScopeIVa, Multimode SPM; Company: Veeco Inc., Santa Barbara, USA), in tapping mode. Calibration was carried out by scanning, using a calibration grid with precisely known dimensions. All scans were performed with commercial Si Nanoprobes SPM tips with a resonance frequency of about 300–330 kHz. During sample preparation, highly diluted aqueous nanofibre suspension was pipetted out and then transferred over freshly cleaved mica surface. It was allowed to dry overnight at room temperature to obtain the thin films.

#### FTIR analysis

The chemical component of the raw wood, alkali treated fibre and nanofibre were analyzed using FTIR spectrometer (Thermo Nicolet Avtar 370). The Infrared spectra of the samples were measured in the range of 400–4000 cm^−1^ with a resolution of 4 cm^−1^ with an interferogram of 32 scans. For the analysis KBr pellet of samples was prepared by mixing 1 mg of the finely ground sample with about 100 mg of the dried KBr powder. A pressure ranges from 69 to 103 Mpa was applied to obtain pellets.

#### TG analysis

TGA analysis of raw wood, chemically treated wood and nanofibre was carried out using Perkin Elmer STA6000. Oven dried samples of 5–10 mg were taken and then placed in a clean ceramic pan. The weight of the pan was nullified initially. The pan with the sample was placed on the TG balance and weighed again. The sample was then heated to 600 °C with a rate of 10 °C min^−1^ under nitrogen atmosphere.

#### XRD analysis

In X-Ray Powder Diffraction Technique (XRD), X-ray equatorial diffraction profiles were acquired by a Bruker D8 Advance diffractometer, equipped with CuKR radiation (λ) (0.1541 nm) to study the relative crystallinity of raw and treated fibers. The fine powdered sample was placed over silica made low back ground sample holder and fixed on the sample stage in the goniometer. The instrument was fixed with a θ–2θ geometry. The voltage and current was 40 mV and 40 mA respectively. The samples were scanned over the 2θ range of 1–90° with a step size of 0.02° and count time of 0.4 s per step. The XRD spectra were acquired from powdered and air-dried samples. The diffractogram was drawn and the intensity and area of crystalline reflections and amorphous background were estimated by the curve fitting method using PeakFit v4.12 software. The degree of crystallinity of the samples was determined by two methods; (1) by using intensity of amorphous and crystalline peaks (Segal peak height method)^80^ and (2) by using area of crystalline fraction and amorphous fraction^81^. The formula used were

1 $${\mathrm{Crystallinity\, index }}\left(\mathrm{CrI}\right)\left(\mathrm{\%}\right) = \frac{[\mathrm{Intensity\, of\, crystalline\, peak }\left(\mathrm{I}002\right)-\mathrm{ Intensity\, of\, amorphous\, peak }\left(\mathrm{I}{\text{am}}\right)] \times 100}{\mathrm{Intensity\, of\, crystalline\, peak }(\mathrm{I}002)}$$

2 $$\mathrm{Crystallinity\, index }\left(\mathrm{CrI}\right)\left(\mathrm{\%}\right) = \frac{(\mathrm{Area\, of\, crystalline\, fraction }\times 100) }{(\mathrm{Area\, of\, crystalline\, fraction }+\mathrm{ Area\, of\, amorphous\, fraction})}$$

### Ethics approval

This study does not contain any experiments with human participants or animals performed by any of the authors. We also confirm that all local, national or international guidelines and legislation were adhered for the use of plants in this study.

## Conclusion

In this work, steam explosion method was adopted to isolate nanofibres from the wood pulp of two hardwood species namely *Eucalyptus tereticornis* and *Casuarina equisetifolia*. The nanofibers obtained in this study had average diameters of 27.801 nm and 28.690 nm respectively for *E. tereticornis* and *C. equisetifolia*. Nanofibres showed high crystallinity, thermal stability and chemical purity. It was interesting to note that the average diameter of nanofibres differed between species though the method and treatment conditions were the same. This study proved that hardwood pulp as raw material and steam explosion as a technology is a viable option for the production of nanofibers of very good properties. There is huge potential for pulp and paper industries to adopt this technology for commercial level production of nanofibres by using the already available infrastructure and resources. Since nanofibres can be used for multiple applications like reinforcement material, nanocarrier etc., its demand is expected to increase multifold in the future.

## Supplementary Information

Supplementary Information.

## Data Availability

The authors confirm that the data supporting the findings of this study are available within the article. The data that support the findings of this study are openly available in the form of Ph.D. thesis at Tamil Nadu Agricultural University, Coimbatore, Tamil Nadu.
