# Supplementary figures and images for: Isolation and characterization of nanocellulose from selected hardwoods, viz., Eucalyptus tereticornis Sm. and Casuarina equisetifolia L., by steam explosion method
Source: Sci Rep. 2023 Jan 21;13:1199. doi: 10.1038/s41598-022-26600-5 (PMC9867748; doi:10.1038/s41598-022-26600-5)

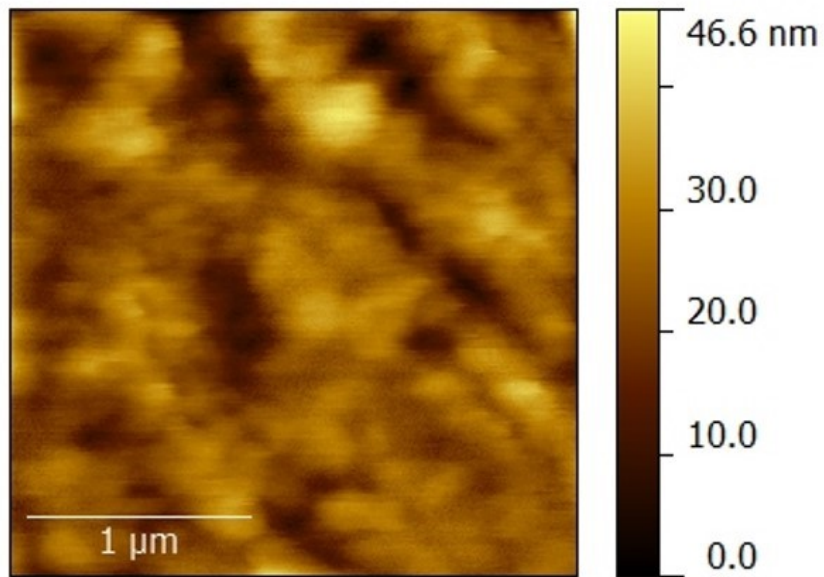

(a)

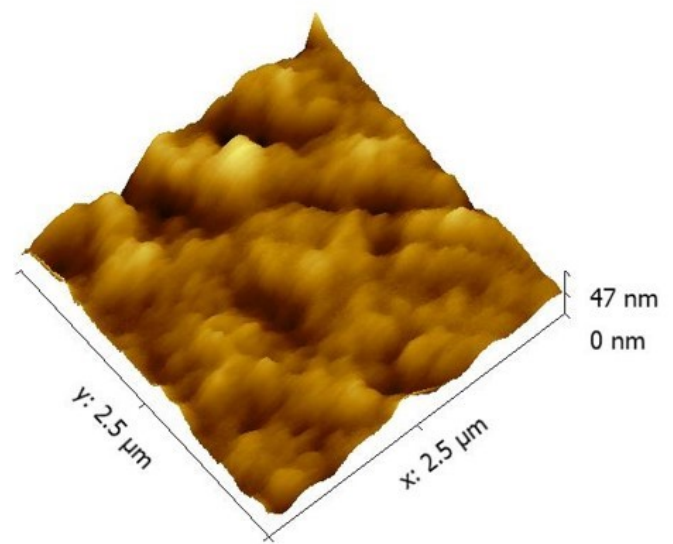

(b)

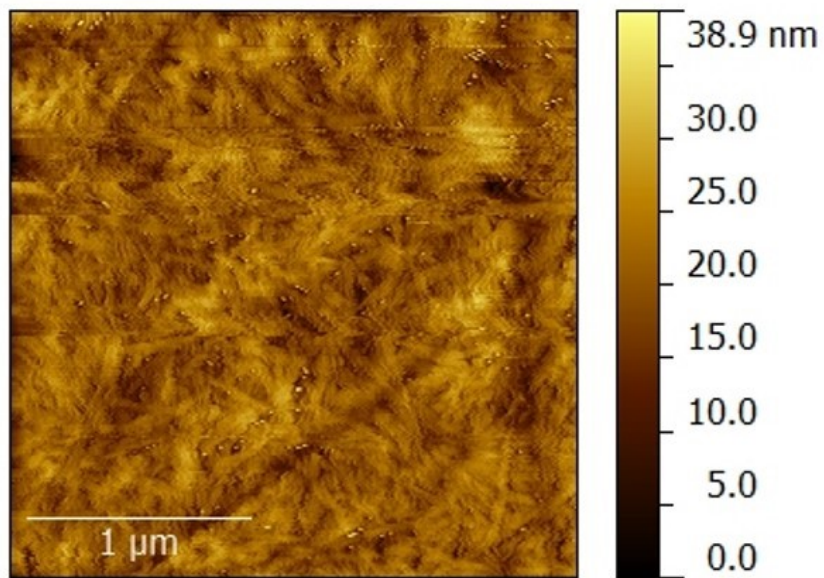

(c)

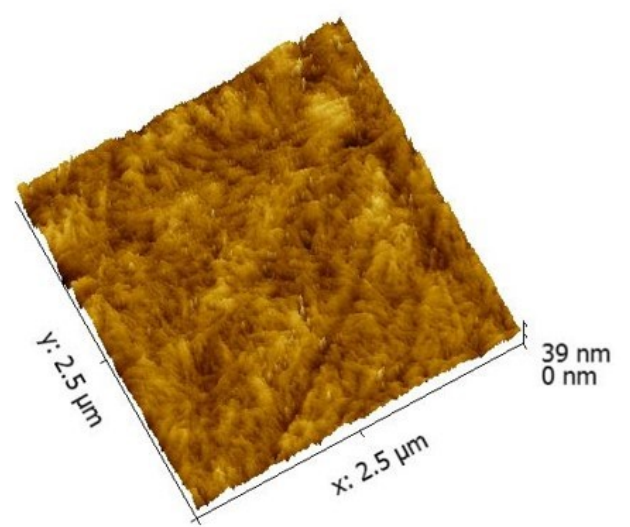

(d)

ESM\_1. AFM images (2D and 3D) of eucalyptus (a and b) and casuarina (c and d) nanofibres

Supplement: Supplementary file 1 — Supplementary Information. [file 41598_2022_26600_MOESM1_ESM.pdf]
